# Development of a Diagnostic Clinical Score for Hemodynamically Significant Patent Ductus Arteriosus

**DOI:** 10.3389/fped.2017.00280

**Published:** 2017-12-22

**Authors:** Annemarie Kindler, Barbara Seipolt, Antje Heilmann, Ursula Range, Mario Rüdiger, Sigrun Ruth Hofmann

**Affiliations:** ^1^Department of Pediatrics, Medizinische Fakultät Carl Gustav Carus, Technische Universität Dresden, Dresden, Germany; ^2^Institute for Medical Informatics and Biometry, Medizinische Fakultät Carl Gustav Carus, Technische Universität Dresden, Dresden, Germany

**Keywords:** arterial duct, clinical diagnostic score, echocardiography, ductus arteriosus Botalli, premature infants, very low birth weight

## Abstract

There is no consensus about the hemodynamic significance and, therefore, the need to treat a persistent ductus arteriosus in preterm newborns. Since the diagnosis of a hemodynamically significant persistent ductus arteriosus (hsPDA) is made by a summary of non-uniform echo-criteria in combination with the clinical deterioration of the preterm neonate, standardized clinical and ultrasound scoring systems are needed. The objective of this study was the development of a clinical score for the detection and follow-up of hsPDA. In this observational cohort study of 154 preterm neonates (mean gestational age 28.1 weeks), clinical signs for the development of hsPDA were recorded in a standardized score and compared to echocardiography. Analyzing the significance of single score parameters compared to the diagnosis by echocardiography, we developed a short clinical score (calculated sensitivity 84% and specificity 80%). *In conclusion*, this clinical diagnostic PDA score is non-invasive and quickly to implement. The continuous assessment of defined clinical parameters allows for a more precise diagnosis of hemodynamic significance of PDA and, therefore, should help to detect preterm neonates needing PDA-treatment. The score, therefore, allows a more targeted use of echocardiography in these very fragile preterm neonates.

## Introduction

The association of hemodynamically significant persistent ductus arteriosus (hsPDA) with intraventricular hemorrhage (IVH), pulmonary hemorrhage, bronchopulmonary dysplasia (BPD), and intestinal complications, such as necrotizing enterocolitis (NEC), is an ongoing field of research and discussed controversially (1–6). There is no consensus about the diagnosis and the hemodynamic significance and, therefore, the need to treat a PDA (7). In preterm neonates, the arterial duct often persists and stays open, due to immaturity, higher prostaglandin levels and hypoxia (8, 9). Due to the change of fetal to postnatal circulation, with a decrease in pulmonary resistance, the blood flow through the patent arterial duct inverses (from a right-to-left into a left-to-right shunt), now leading to a pulmonary overflow and a steal-phenomenon of the systemic circulation. This leads to hemodynamic instability with the following clinical symptoms: respiratory deterioration (RD) (due to increased lung perfusion), increased precordial pulsations, tachycardia, hepatomegaly (HM), accentuated femoral pulses (FP) (due to the ductal steel phenomenon and increased cardiac charge) and oliguria and metabolic acidosis (AC) (due to decreased systemic perfusion).

Since untreated hsPDA alters organ perfusion and potentially induces hypoxia in these organs (e.g., brain, kidney, bowels), several studies have investigated the ability of biomarkers to discriminate between infants with and without hsPDA. Natriuretic peptides (such as BNP and NTpBNP) showed a strong predictive ability for early detection of an hsPDA (on days 3 and 4) in preterm infants (10, 11). In another clinical study, ischemia-modified albumin (IMA) was suggested as a new biochemical marker for the diagnosis and monitoring of a successful treatment of hsPDA in premature infants (12). A positive correlation was found between IMA and PDA diameter, and LA/Ao ratio (12). However, further studies evaluating the diagnostic accuracy of natriuretic peptides and IMA for detection of hsPDA showed wide variability according to assay criteria and patient characteristics (13). Therefore, generalizability is limited and further studies with standardized methods as well as objective clinical and echocardiography parameters are needed.

In recent years, fewer medical interventions and more supportive measures such as fluid restriction (5, 14) and non-invasive ventilatory support have been implemented to reduce the occurrence of hsPDA. In a recent publication, extremely preterm infants between 23–26 weeks with an hsPDA (defined as “left-to-right flow by gain-optimized color Doppler” measuring ≥2 mm) requiring ventilator treatment were divided in two groups, “mandatory closure” with i.v. indomethacin and/or surgical ligation versus “non-intervention approach” (fluid restriction and diuretics with respiratory support as needed) (15) and compared regarding there outcome. Surprisingly, the non-intervention group was reported with significantly less BPD despite longer PDA exposure and was not associated with increased mortality or morbidities such as IVH or NEC (15). This result could perhaps partly be explained by the high rate of surgical ligation (82%) in the “mandatory treatment” group, leading to a higher rate of postoperative myocardial dysfunction and successively higher use of inotropic drugs (15). A longer phase of positive pressure ventilation support in these patients as an additional reason for the higher rate of BPD in the “mandatory treatment” group could be excluded (15). However, looking at their data, the duration of supplemental oxygen was significantly higher (*p* < 0.05) in the “mandatory treatment” group. At this point, one could speculate that the oxygen induced initiation of inflammatory processes within the lung could play a role for the higher BPD rate in this group. Another speculation raced by the authors is the influence of more prolonged use of high-frequency oscillatory ventilation in the “non-intervention approach” group, which perhaps may have contributed to the lower BPD rate in this group (15).

However, despite the mentioned conservative measures there are still patients who develop hsPDA. But, whether and when such infants require treatment is still controversially discussed (16). Therefore, prospective randomized clinical trials of current pharmacological treatment “standards” (such as i.v. ibuprofen or i.v. indomethacin) versus “non-intervention support” are needed to answer the question, whether hsPDA contributes to the development of other co-morbidities such as BPD, NEC, retinopathy of prematurity, and IVH. In addition, there is a need for clearly defined and widely applicable clinical and echocardiography criteria for the selection of patients with clinical relevant hsPDA who may benefit from ductul closure.

The aim of this study was to evaluate the reliability of a clinical score to predict the hemodynamic significance of the PDA, which should help to decide whether to perform an echocardiography.

## Patients and Methods

### Study Population

This single-center observational cohort study was performed in a level one neonatology center. A total of 200 neonates with a gestational age (GA) of ≤31 weeks admitted between October 2008 and September 2010 was screened for eligibility. 47 patients had to be excluded from the study because of lacking datasets. Therefore, 154 preterm neonates (59.7% male, 40.3% female) were included in the study population.

### Ethical Approval

All procedures performed in studies involving human participants were in accordance with the ethical standards of the institutional and/or national research committee and with the 1964 Helsinki declaration and its later amendments or comparable ethical standards. Permission for this study was approved by the hospital’s Human Ethics Research Committee (EK359112010).

### Informed Consent

For this type of study informed consent was not required since all clinical and echocardiography examinations were routine care, which has been signed by the parents.

### Echocardiographic Criteria

*Echocardiographic criteria* indicating hsPDA were ductus arteriosus diameter at its narrowest part >1.5 mm (17, 18) and at least one additional criteria such as absent (end-)diastolic flow in the celiac trunk (19, 20), maximal velocity of the left-to-right shunt through the PDA < 1.5 m/s (20, 21), left atrial-to-aortic root ratio >1.3 (19, 22–27).

### Data Collection

The clinical score to detect an hsPDA was based on a score described by Kabus (28). The original score was modified by deleting X-ray-results and including new clinical criteria. The new scoring system consists now of the following eight clinical criteria: pulsations of the precordium (PP), systolic murmur (SM), tachycardia [heart rate (HR)] > 160 bpm, apnea or mechanical ventilation (A/MV), bounding FP, HM, metabolic AC with pH <7.30, and base excess <−5 and RD, indicated by increased oxygen supplementation, increased non-invasive or invasive respiratory support, increased apnea frequency and mounting hypercapnia. For each criterion that was present, one point was scored. If the score reached ≥2 points, an hsPDA was suspected and echocardiography was performed. The score was assessed daily by the attending neonatologist, starting with day 2 until day 10 of life.

**Figure 1:**
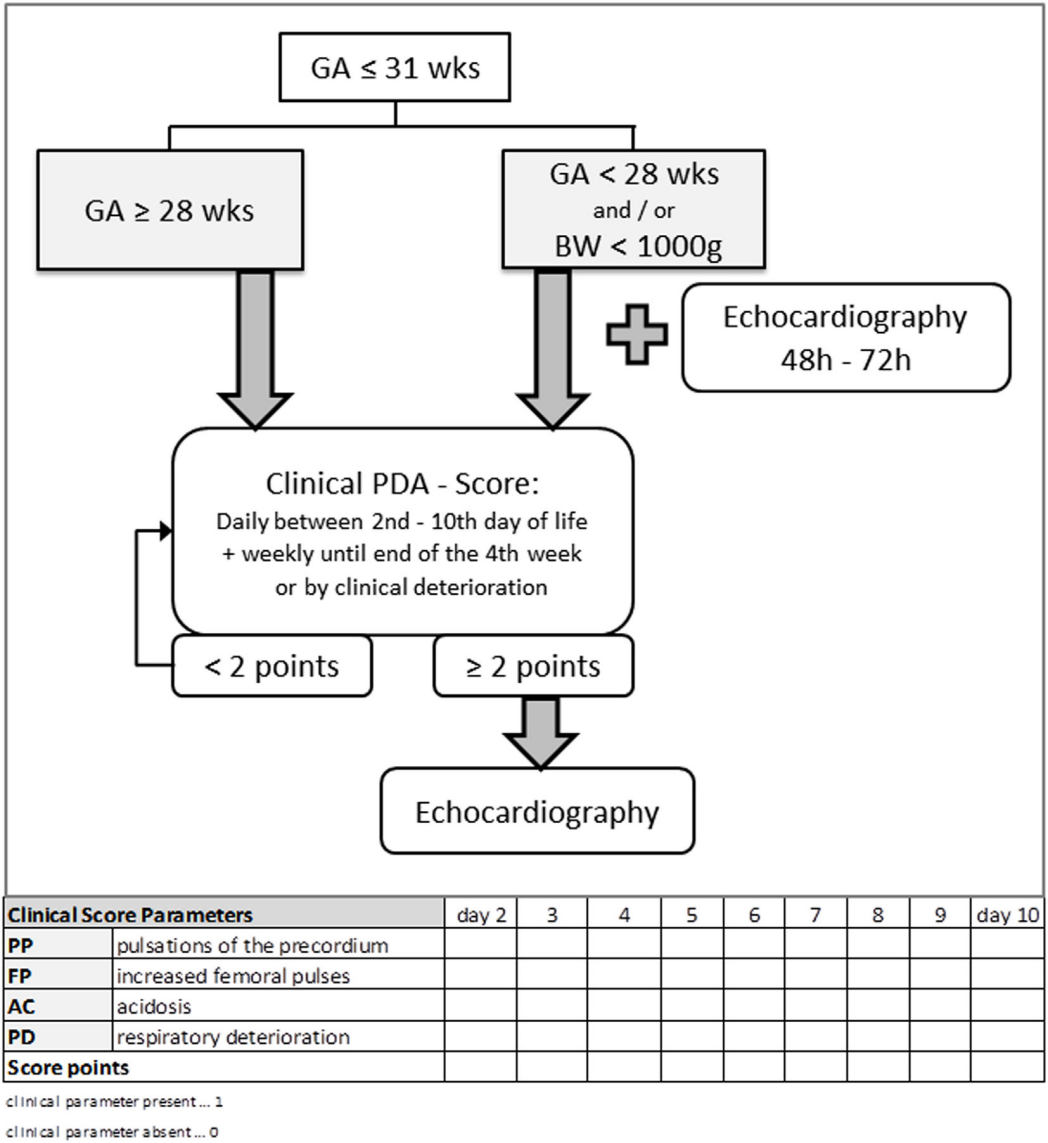
Flowchart of the clinical approach to diagnose a clinical relevant hemodynamically significant persistent ductus arteriosus (hsPDA) using clinical parameters and echocardiography. The scoring tool was used in all premature neonates equal or less than 31 weeks of gestation. In all preterm infants GA <28 weeks or BW <1,000 g, an initial echocardiography was performed between 48 and 72 h of life, in addition to the clinical score. All other premature neonates GA ≥28 weeks were assessed by clinical scoring and received an echocardiography only if the score was ≥2 points. GA, gestational age; BW, birth weight.

The scoring tool was used in all premature neonates equal or less than 31 weeks of gestation. In all preterm infants, GA < 28 weeks or birth weight (BW) < 1,000 g, an initial echocardiography was performed between 48 and 72 h of life, in addition to the clinical score. All other premature neonates GA ≥28 weeks were assessed by clinical scoring and received an echocardiography only if the score was ≥2 points (Figure 1).

### Statistical Analysis

Statistical analyses were performed using IBM SPSS 22.0 (IBM, Chicago, IL, USA). For nominal scaled parameters, absolute and relative frequency was determined. Correlations between two or more nominal scaled parameters were examined using the chi-square-test (or the exact Fisher test). For that, observed versus expected frequencies were compared by cross-tabulation tables. A *t*-test was used for metric scaled variables and independent samples (normal distribution). *p*-Values < 0.05 were considered statistically significant. For comparing the quality of combinations of particular features, receiver operating characteristic (ROC) (29) curves were used to determine the sensitivity and specificity by calculating the area under the curve (dependent on the determined cutoff level).

## Results

### Demographic Data of the Study Population

The mean GA of the study population (*n* = 154) was 28.1 weeks (SD ±2.2), with a mean gestational weight (GW) of 1,052 g (SD ±374). Additional demographic data are shown in Table 1, and risk factors for the development of an hsPDA and the outcome of the study population regarding chronic sequelae are shown in Table S1 in Supplementary Material. None of our patients had major associated malformations.

**Table 1:** Demographic data of the study population.

Demographic data		SD (±)
Study population (total patients, % females, % males)	154 (40.3, 59.7)	
Mean gestational age (weeks)	28.1	2.2
Mean gestational weight (g)	1,052	374
Mean APGAR 1 min	4	2.2
Mean APGAR 5 min	7	1.7
Mean APGAR 10 min	8	1.2
Mean umbilical artery pH	7.29	0.12
Mean duration of hospitalization (days)	71.9	35.9
Mean BW (g) at discharge	2,405	475
Preterm neonates needing respiratory support (%)	96.1	
Mortality (%)	7.1	

An hsPDA was suspected according to clinical scoring in 86% of infants between the second and fourth day of life (mean 3.15 days). Out of the 154 included preterm neonates, 88 patients (57.1%) were diagnosed with hsPDA by the gold standard of echocardiography. The incidence of hsPDA in preterm neonates below 28 weeks was significantly higher (74.6%) compared to preterm neonates between 28–31 weeks (43.7%; *p* < 0.001).

### Evaluation of the Clinical PDA Score

To evaluate the clinical PDA score including eight parameters (PP, HR, A/MV, FP, SM, HM, AC, PD) we compared its sensitivity and specificity to predict the presence of an hsPDA as diagnosed by echocardiography. Using logistical regression analysis and ROC (29) curves, we compared the strength of combining particular items of the score and determined its sensitivity and specificity. More precise, for each day (2–4) we performed logistical regression analyses of all eight clinical parameters (from the initial score) and tested for statistical significance. Parameters with *p* < 0.1 were used for the “new short clinical score.” Four parameters were included in the final model. With these four parameters we performed ROC analyses for comparing the quality of combination of the particular parameters for each day to predict the development of hsPDA. With the coordinates of the ROC curves, we determined the best cutoff-value, which has been 1.5. With that we calculated specificity and sensitivity for the clinical score.

**Figure 2:**
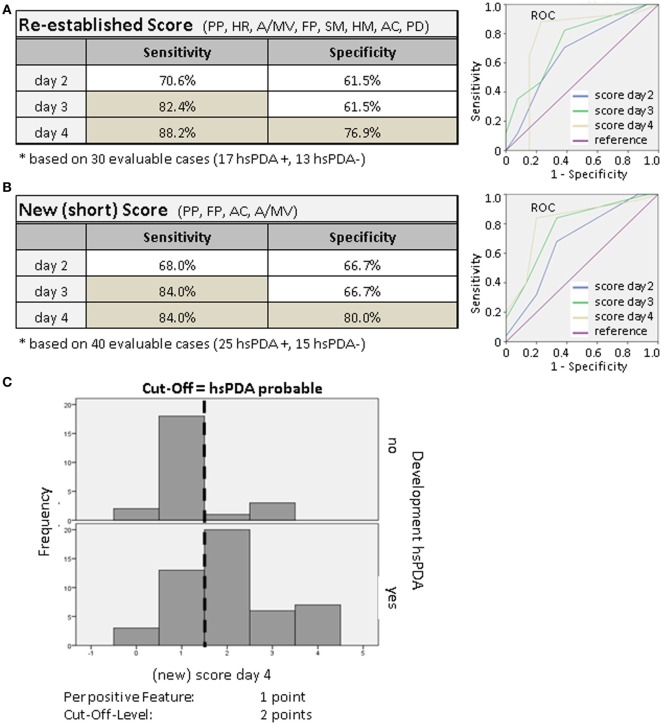
**(A,B)** Sensitivity and specificity of the clinical PDA score. Using multivariate logistic regression analysis (daily) and receiver operating characteristic (ROC) (29) curves, we compared the power of combinations of particular features and determined the sensitivity and specificity (dependent on the determined cutoff-level). The theoretical optimal cutoff-level was 1.5 points. **(C)** Screening of the validity of the clinical score. We tested to see if the score of ≥2 points was able to discriminate between the two groups with and without hsPDA. For the presence of a parameter we increased the number of points from 1 to 2 or 3. The frequency of the given points at day 4 of the score is shown as an example. On the upper part the infants without hsPDA are shown, those with hsPDA are shown on the lower part. Newborns with hsPDA usually reached a score of 2 or more points.

As shown in Figure 2A, the sensitivity and specificity of this score increased from day 2 to day 4, and reached 88.2% sensitivity and 76.9% specificity.

### Score Parameters and Their Significance

To weight single score citeria, cross-tabulation tables were used to contrast the single parameters with the development of an echocardiographic confirmed hsPDA (univariate analysis). By determination of the adapted residuum and the exact significance by the Fisher test, the significant and strongest statistical parameters were determined.

The test to summarize clinical parameters by logistic regression analysis allowed us to specify the following significant symptoms: pulsations of the precordium, bounding FP, apnoe/mechanical ventilation and metabolic AC (Table 2). On days 3 and 4 of life these parameters correlate most strongly with the development of an hsPDA verified by echocardiography.

**Table 2:** Clinical PDA score parameters.

Significant clinical parameters									
Day of life	2	3	4	5	6	7	8	9	10
PP	1.0	**0.027**	**0.026**	0.120	0.738	1.0	0.171	0.292	1.0
HR	0.261	1.0	0.589	0.204	0.168	1.0	1.0	0.706	1.0
A/MV	0.698	**0.033**	0.782	**0.029**	0.098	0.715	0.201	0.610	1.0
FP	0.357	**0.038**	**0.021**	0.186	0.159	0.244	1.0	0.542	1.0
SM	0.761	0.221	0.061	0.339	0.339	0.552	1.0	0.542	0.269
HM	–	–	1.0	1.0	–	–	–	–	0.185
AC	**0.030**	**0.005**	**0.033**	0.401	**0.039**	0.055	0.057	0.121	1.0
PD	1.0	0.609	1.0	1.0	1.0	1.0	1.0	0.102	0.318

*Significance of clinical parameters compared to the diagnosis of hsPDA by echocardiography. p-Values < 0.05 were considered statistically significant*.

*PP, pulsations of the precordium; HR, heart rate; A/MV, apnea or mechanical ventilation; FP, femoral pulses; SM, systolic murmur; HM, hepatomegaly; AC, acidosis; PD, pulmonary deterioration*.

### New Short Clinical PDA Score

With the aim to simplify this clinical scoring system further, we tried to reduce the number of parameters by using only the previously tested statistically significant parameters in a so-called new (short) PDA score. By again using logistical regression analysis and ROC curves, we compared the power of combinations of these parameters and determined the sensitivity and specificity. The new short clinical PDA score reached a calculated sensitivity of 84% and a specificity of 80% on day 4 (Figure 2B), which is similar to the re-established initial score.

### Validity of the Clinical Score

In order to further increase sensitivity and specificity, we gave single score items a different weight. For the presence of a parameter, we increased the number of points from 1 to 2 or 3. But this did not improve the validity of the score (not shown). In Figure 2C, the distribution (frequency) of the given score points at day 4 is shown as an example. On the upper part the infants without hsPDA and on the lower part the ones with hsPDA are shown. Newborns with hsPDA usually reached a score of 2 or more points. The cutoff-level of the clinical score stayed at 2.

## Discussion

Persistent ductus arteriosus is a physiological phenomenon of the preterm infant with a delayed closure during the first week of life. In preterm neonates below 28 weeks, who often need ventilatory support, the prevalence of ductal patency at 48–72 h of life is still high [about two-thirds (30, 31)], and varies between centers (32). There is a higher incidence of hsPDA in small for GA infants (33, 34).

In accordance with published data (35), the frequency of hsPDA in our study population is inversely related to GA of preterm infants. In addition, preterm neonates initially supported by continuous positive airway pressure were significantly less likely to develop an hsPDA compared to initially mechanically ventilated preterm neonates [also reported by Ref. (32)].

Prior to the routine use of echocardiography in neonatology, clinical symptoms such as bounding pulses, a hyperactive precordium, increased blood pressure amplitude, SM, as well as an increased cardiothoracic ratio on the chest X-ray were used for diagnosing PDA (36–38). Furthermore, the arterial duct was usually searched for when the infant could not be weaned from the respirator or cardiac failure occurred.

At present, echocardiography is the gold standard for the diagnosis of hsPDA, but there is not just one criterion confirming this diagnosis. It is rather a summary of several echo-criteria in combination with the clinical deterioration of the preterm neonate allowing to confirm this diagnosis (Table S2 in Supplementary Material). However, clinical and echocardiography criteria are not uniform between centers.

Despite the general meaning that neonatal echocardiography can be performed easily and quickly by an experienced specialist, this examination needs opening of the incubator with potential risk of hypothermia, needs several sectional planes and doppler measurements and is potentially disturbing and stressing for extremely low BW infants below 28 weeks (or < 1,000 g). For this reason, ultrasound examinations should be performed only if necessary. Therefore, a clinical score for the diagnosis of hsPDA potentially reduces the number of ultrasound examinations in such small, fragile and immature neonates.

Here, we investigated to what extent a new clinical scoring system for the detection of hsPDA is able to predict the hemodynamic significance of the PDA as diagnosed by echocardiography. We tested combinations of eight clinical parameters compared to hsPDA-diagnosis by echocardiography. As a result, a new scoring system composed of four significant symptoms (pulsations of the precordium, bounding FP, apnea/mechanical ventilation, and metabolic AC) was established. The new score reached a calculated sensitivity (84%) and specificity (80%). For the parameter A/MV, we adapted our clinical criteria to “pulmonary deterioration” compared to the previous day, as indicated by increased oxygen supplementation, increased non-invasive or invasive respiratory support, increased apnea frequency and mounting hypercapnia.

To avoid over-diagnosis of hemodynamically non-significant (irrelevant) PDA and to reduce the number of unnecessary ultrasound examinations in premature neonates, an initial standard echocardiography was only performed in preterm neonates below 28 weeks or BW < 1,000 g between 48 and 72 h of life (in addition to the clinical score). All premature neonates GA < 31 weeks were assessed by the clinical scoring system during the first week of life and in some cases further, and received an echocardiography only if the score was ≥2 points. To evaluate the success of this approach to reduce echocardiography numbers is an ongoing investigation for the new scoring system composed of four significant symptoms.

To determine the need for treatment in preterm neonates with hsPDA standardized protocols or scoring systems for the echocardiography are lacking, and the comparability and repeatability of echocardiography in preterm infants with suspected PDA is far from optimal. This newly established clinical score provides the potential to evaluate a standardized echocardiography approach for diagnosing hsPDA. A follow-up cohort study to address this issue in preterm infants below 28 weeks is actually on the way. Therefore, this clinical score is developed as an addition to echocardiography to find more uniform criteria for the diagnosis of hsPDA, not to replace it.

The limitations of the presented study are the non-controlled observational study design and the variable echocardiography criteria between different neonatal care centers. Prospective controlled trials are needed to establish standardized protocols and robust echocardiography criteria for the diagnosis of hsPDA and the need for treatment.

In conclusion, this new clinical PDA score is non-invasive, easy to use and quick to implement. The calculated score-sensitivity and specificity is 84 and 80% (on day 4), respectively. Despite the most predictive value of the clinical score for the development of hsPDA between days 3 and 4, the score can be used from day 2 on up to clinical stabilization (without needing respiratory support). Since the diagnosis of hsPDA is made by a summary of several echo-criteria in combination with the clinical deterioration of the preterm neonate, this score allows for a more standardization in confirming this diagnosis. The continuous assessment of these clinical parameters by using the PDA score also allows for a more targeted use of echocardiography, and might help to reduce the number of ultrasound examinations in preterm neonates.

## Ethics Statement

All procedures performed in studies involving human participants were in accordance with the ethical standards of the institutional and/or national research committee and with the 1964 Helsinki declaration and its later amendments or comparable ethical standards. Permission for this study was approved by the hospital’s Human Ethics Research Committee (EK359112010).

## Author Contributions

AK collected data and performed analysis and critically revised content of manuscript. BS collected data and critically revised content of manuscript. AH collected data and critically revised content of manuscript. UR helped with the statistical analysis of the data and critically revised content of manuscript. MR supported conceptualizing study design and critically revised content of manuscript. SH collected data, wrote the manuscript, and conceptualized the study.

## Conflict of Interest Statement

The authors declare no conflicts of interest. This research did not receive any specific grant from funding agencies in the public, commercial, or not-for-profit sectors.

## Abbreviations

AC, acidosis; AIS, amnion infection syndrome; A/MV, apnea or mechanical ventilation; BPD, bronchopulmonary dysplasia; BW, birth weight; CPAP, continuous positive airway pressure; FP, femoral pulses; GA, gestational age; HM, hepatomegaly; HR, heart rate; hsPDA, hemodynamically significant persistent ductus arteriosus; IVH/ICH, intraventricular hemorrhage/intracerebral hemorrhage; NEC, necrotizing enterocolitis; PD, pulmonary deterioration; PI, postnatal infection; PHT, pulmonary hypertension; PP, pulsations of the precordium; PRM, premature rupture of the membrane; PVL, periventricular leukomalacia; RDS, respiratory distress syndrome; ROC, receiver operating characteristic; ROP, retinopathy of prematurity; SM, systolic murmur; wks, weeks of gestational age.
